# Assessing small-lesion detectability and acquisition time optimisation in silicon-detector-Based PET: a phantom study

**DOI:** 10.1186/s40658-025-00821-9

**Published:** 2025-12-28

**Authors:** Nicholas Leybourne, Vineet Prakash, Mohammad Hussein, Andrew Fenwick, Peter Strouhal, Philip Evans, Lucia Florescu

**Affiliations:** 1 Centre for Vision, Speech and Signal Processing, University of Surrey, Stag Hill, Guildford, GU2 7XH Surrey Northern Ireland, UK; 2 National Physical Laboratory, Department, Hampton Road, Teddington, TW11 0LW Middlesex UK; 3 Department of Nuclear Medicine, Royal Surrey NHS Foundation Trust, Egerton Road, Guildford, GU2 7XX Surrey UK; 4 Alliance Medical Ltd, Iceni Centre, Warwick Technology Park, Warwick, CV34 5AH Warwickshire UK

**Keywords:** PET/CT imaging, Silicon photomultipliers, Phantom imaging, Small-lesion detection, Image quality, Scanner performance, Acquisition time reduction

## Abstract

**Background:**

The adoption of silicon photomultiplier (SiPM) detectors over conventional photomultiplier tubes (PMTs) in Positron Emission Tomography (PET) has enhanced overall system performance. In this phantom study, small-lesion detectability was assessed for SiPM-based and PMT-based PET systems for various inhomogeneity sizes, acquisition times and activity contrasts between the inhomogeneity and background.

**Methods:**

Six spheres of internal diameters ranging between 4.0 mm and 13.0 mm were integrated into a NEMA/IEC PET Body Phantom and filled with fluorodeoxyglucose, with a sphere activity concentration of 29.2 MBq/L and five sphere-to-background activity concentration ratios between 4 and 20. Scans were performed with an SiPM-based system and a PMT-based PET system for each sphere-to-background activity concentration ratio for acquisition times between 1 and 10 min, and image reconstruction was performed with QClear for both systems. Reconstructed images were evaluated for lesion detectability by a lesion detectability index, contrast-to-noise ratio and lesion detectability Likert scales with validation by comparison with the Rose criterion. A model to estimate the acquisition time for each sphere to be detectable was derived and acquisition time was compared.

**Results:**

The SiPM-based system demonstrated superior lesion detectability, identifying smaller and less active spheres with shorter acquisition times. For a sphere-to-background activity concentration ratio of 10 and a sphere internal diameter of 6.2 mm, the SiPM-based system achieved a contrast-to-noise ratio of 15.8 and a lesion detectability Likert score of 3, compared to 12.0 and 2, respectively, for the PMT-based system. The acquisition time of the SiPM-based system could be reduced by between 1.6% and 89%, depending on sphere size and sphere-to-background activity concentration ratio. The minimum CNR required for a sphere to achieve a detectability Likert score of 0.5 was 6.3, consistent with the Rose criterion.

**Conclusion:**

SiPM-based PET has enhanced lesion detectability, especially for smaller, less active regions and for shorter acquisition times. A five-point Likert scale is an effective measure of lesion detectability. Guidance is also provided for choosing the acquisition time as a function of lesion size and activity uptake, and for changes in image quality testing protocols.

## Introduction

Positron emission tomography (PET) is an important imaging tool for the detection and management of cancer, but has typically faced challenges in achieving optimal image quality, particularly in terms of spatial resolution and sensitivity. These factors are critical as they directly influence diagnostic accuracy and subsequent treatment decisions [1–4]. With advancements in PET detector technology, there has been a notable shift from conventional photomultiplier tubes (PMTs) to silicon photomultipliers (SiPMs) [5, 6]. To fully realise the clinical potential of SiPM-based systems, it is important to assess their benefits in terms of image quality. In particular, it is often challenging to detect small lesions with PMT-based PET, and SiPM-based systems have shown great promise for new clinical applications in this area [7, 8]. Furthermore, current guidelines, such as the NEMA NU 2-2018 [9], focus on image quality for lesions of 10 mm or larger. However, the potential of modern PET technology to detect smaller lesions necessitates an updated approach that includes assessing image quality for volumes under 10 mm in diameter [8, 10].

Studies comparing SiPM-based and PMT-based PET systems have consistently demonstrated that SiPM-based PET systems offer superior image quality [6, 11–15] for lesions larger than 10 mm. However, for smaller lesions the results are limited. Furthermore, studies widely report limitations such as variable radionuclide uptake in clinical situations and the use of phantom spheres larger than 10 mm in phantom studies, citing the necessity for further evaluation [8, 10, 12]. These, therefore, underscore the need for dedicated studies to accurately assess the performance of SiPM-based PET systems in detecting sub-10 mm lesions in various clinical situations, to enable these technological advancements to translate into meaningful clinical improvements.

Only a limited number of studies have comparatively assessed SiPM-based PET for small-lesion detectability. Kersting et al. [16] used small spheres with internal diameters ranging between 3.7 and 9.7 mm to compare the performance of a SiPM-based Biograph Vision PET/CT system and a PMT-based Biograph mCT PET/CT system for Iodine-124 (^124^I) lesion detectability. The study found that the SiPM-based system could detect lesions smaller than 6.5 mm which were not visible on previous-generation systems and activity concentration could be reduced by a factor of 0.52.

Satoh et al. [14] compared quantitatively and qualitatively the image quality between a SiPM-based and a PMT-based system using a phantom with four spheres of internal diameters 3.0 mm, 5.0 mm, 7.5 mm and 10.0 mm. It was found that the SiPM-based system was superior to the PMT-based system in terms of sharpness, smoothness and detectability but also noted that the PMT-based system may have better ability in detecting lesions at the edges of the field-of-view and when activity in the background is high.

Oddstig et al. [17] used a NEMA IEC Body Phantom and a Jaszczak Phantom with small spheres with internal diameters ranging between 4.0 mm and 14.4 mm to assess a PMT-based Discovery D690 (D690) PET/CT system and a SiPM-based Discovery MI (DMI) PET/CT system (GE Healthcare, Milwaukee, WI, USA) for identical acquisition and reconstruction protocols. The study found only marginal differences in the ability to detect small hotspots between the two scanners and suggested that the improved image quality reported with the SiPM-based PET system is likely more attributable to the advanced reconstruction algorithms rather than the hardware improvements alone. Adler et al. [18] used a phantom with 7 spheres with internal diameters ranging between 4.0 mm and 15.4 mm to assess 5 different PET/CT systems (1 SiPM-based, 4 PMT-based) with varying sphere-to-background activity concentration ratios (SBRs) of 1.88 to 15, varying voxel size of 1 mm to 4 mm and varying acquisition times of 1 to 16 min. No notable benefits to lesion detectability were found for the SiPM-based system. Other studies assessed the feasibility of shorter acquisition times for the same image quality and lesion detectability using a SiPM-based PET system. It was found that a reduction of $75\%$ [19], and a reduction from 180-210 s/bed to 90 s/bed [20], was possible, suggesting that SiPM-based PET systems offer improved lesion detectability. Based on these studies, there is therefore no clear consensus on the benefits of SiPM-based PET for small-lesion detectability and further qualitative investigation is needed. In particular, a single reduction in acquisition time fails to consider variations in lesion size and radionuclide uptake, and a more effective method is needed to estimate acquisition time reductions that depend on the diverse clinical variables.

The objective of this study was to qualitatively and quantitatively compare the performance of SiPM-based and PMT-based PET systems in detecting small lesions using small phantom inserts with varying radioisotope uptake relative to the background and various acquisition times, for the same reconstruction method. This study also introduces a model to estimate the acquisition time required to achieve a specified lesion detectability for a given lesion size and activity uptake, which is used to estimate the scan time saving achievable with the SiPM-based system.

## Methods

### PET/CT systems

A GE Discovery 710 (D710) PET/CT system (GE Healthcare, Milwaukee, WI, USA) and a GE Discovery MI Gen 2 5-ring (DMI Gen2) PET/CT system (GE Healthcare, Milwaukee, WI, USA) were used for imaging in this study. Both systems employ lutetium–yttrium oxyorthosilicate (LYSO) scintillation crystals but the D710 is a PMT-based system whereas the DMI Gen2 is SiPM-based. The full specifications for these systems have been described elsewhere [21–24] and are summarised in Table 1.

**Table 1:** Hardware characteristics of the GE Discovery 710 and GE Discovery MI Gen 2 5-ring PET/CT systems

PET/CT system	GE Discovery 710	GE Discovery MI
Axial field of view (mm)	157	250
Crystal size ($\hbox {mm}^3$)	4.2 $\times$ 6.3 $\times$ 25.0	3.95 $\times$ 5.3 $\times$ 25.0
Crystal array per block	9 $\times$ 6	4 $\times$ 9
Detector ring diameter (mm)	810	744
Number of detector rings	24	36
Slice thickness (mm)	3.27	2.80
Number of detector blocks	256	680
Number of individual crystals	13824	24480
Time of flight resolution (ps)	500	390

### Phantom

**Fig. 1:**
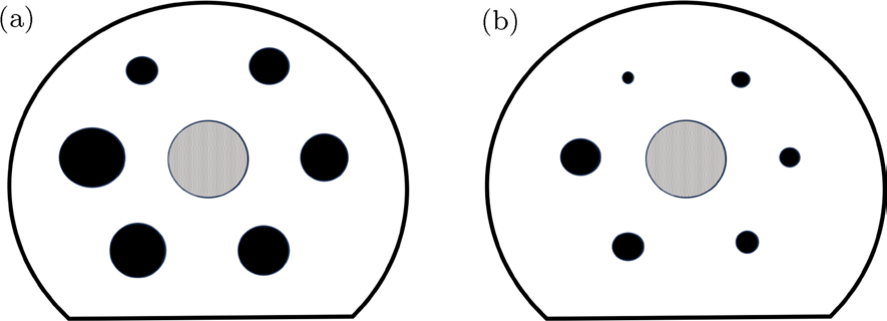
Central plane of phantoms with central lung-mimicking insert (grey) and spheres (black) for the standard NEMA phantom (**a**), and the modified NEMA phantom (**b**)

The NEMA IEC Body Phantom (Data Spectrum Corporation, Durham, NC, USA) contains six holes for the insertion of hollow spheres with coplanar centres which can be filled with radiotracers. Commonly, spheres with internal diameters of 10.0 mm, 13.0 mm, 17.0 mm, 22.0 mm, 28.0 mm and 37.0 mm are used. For this work, the four largest were replaced with the ECT/MI-HS/SET4 (Data Spectrum Corporation, Durham, NC, USA) sphere set of internal diameters of 4.0 mm, 5.0 mm, 6.2 mm and 7.9 mm. To mimic lung attenuation, a Styrofoam insert with an average density of 0.3 g/cm^3^ was placed in the centre of the phantom. A schematic of a standard NEMA phantom [25] and the modified phantom used in this work is presented in Fig. 1.

### Data acquisition and image reconstruction

Fluorodeoxyglucose (^18^F-FDG) was mixed with deionised water and injected into the spheres and background of the phantom and scanned at an initial SBR of 20. Activity concentration in the spheres was 29.2 MBq/L and 34.6 MBq/L for the first scan for the SiPM-based and PMT-based system, respectively. In our experiments, uncertainty in activity measurements was 3%. Further scans were then taken with activity added to the background of the phantom after each scan, resulting in SBRs of 20, 15, 10, 8 and 4. These values were chosen to simulate tumour-to-background activity concentration ratios commonly seen in clinical practice [17, 26]. A table of activity concentrations in the spheres and phantom background, and the achieved SBR are presented for both systems in Supplementary Table 1 for each target SBR. The actual SBR percentage difference between SiPM- and PMT-based systems, derived from measured activities in the spheres and background, were minimal at under 1% for most target SBRs. However, target SBRs of 10 and 20 showed higher percentage differences of 6.27% and 2.82%, respectively. The phantom was positioned such that during scanning the centres of the inserts appeared in the same central slice of the resulting image. To account for the difference in sphere activity concentration between the SiPM-based and PMT-based systems and to ensure an equivalent time-activity product between the two systems, the acquisition times for the PMT-based system were adjusted as [17, 27]

1 $$\begin{aligned} T_1 = \left( \frac{A_2}{A_1}\right) T_2, \end{aligned}$$

where $T_1$, $A_1$ and $T_2$, $A_2$ are the acquisition time and activity concentration for the PMT-based and SiPM-based systems, respectively.

Image reconstruction was performed with QClear (GE Healthcare, Milwaukee, WI, USA), a Bayesian penalized-likelihood reconstruction algorithm for PET [28]. This requires a single user-defined parameter, $\beta$, which based on clinical standards was set to 600 [28]. The reconstructed image matrix size was 256 × 256 with a pixel size of 2.7 mm, and 128 × 128 with a pixel size of 5.5 mm, for the SiPM-based and PMT-based systems, respectively. These were chosen based on the clinical standard for each system [17, 29]. All reconstructions included CT-based attenuation correction and were performed using on-scanner software. For each SBR, the phantom was scanned for a total of 10 min, in one bed position. Using the data acquired for a scanning time of 10 min, images were reconstructed for additional acquisition times of 1, 2, 3, 4, 5, 6, and 8 min for both systems.

### Image analysis

The quality of the reconstructed images was assessed for the central slice of each scan in terms of the contrast-to-noise ratio (CNR),

2 $$\begin{aligned} CNR = \frac{\overline{S} - \overline{B}}{\sigma _{B}}, \end{aligned}$$

lesion detectability index (LDI),

3 $$\begin{aligned} LDI = \frac{A_{M} - \overline{B}}{\sigma _{B}}, \end{aligned}$$

and contrast recovery (CR),

4 $$\begin{aligned} CR = \frac{A_{M}}{A_{0}}, \end{aligned}$$

where $\overline{S}$, $\overline{B}$ are the mean reconstructed activity of the spheres and background, respectively, $\sigma _{B}$ is the standard deviation of the reconstructed activity of the background, and $A_{M}$ and $A_{0}$ are the maximum reconstructed activity concentration and actual activity concentration in each sphere, respectively. $A_{0}$ was measured during sphere filling. Uncertainty of $\overline{S}$, $\overline{B}$ and $A_{M}$ was quantified as the standard deviation of the respective voxel values within the considered domain (sphere or background). Uncertainty of CNR, LDI and CR was calculated by adding in quadrature the uncertainties of the quantities in their respective equations. The uncertainties of CNR, LDI and CR are small (around 1%) and thus the error bars are not visible in the figures representing these quantities. Lesion detectability was assessed using a three-point and five-point Likert scale [30] which was developed and scored with supervision from a consultant radiologist. The five-point Likert scale was compared with the three-point Likert scale as 5-point Likert scales typically determine lesion detectability with a finer resolution than a three-point Likert scale [18, 31]. Tables 2 and 3 present the definition of each point on the Likert scales. To correlate the subjective Likert scale with the objective CNR, an extended CNR was chosen for assessment against the Likert scales to account for the fact that the spheres are not point sources and therefore the shape and size of each sphere must be considered. This was selected based on previous studies [32, 33] and calculated as

5 $$\begin{aligned} CNR_E = CNR \times \sqrt{N} \times CR, \end{aligned}$$

where N and CR represent the number of voxels within the ROI and contrast recovery, respectively, for a given sphere. The average extended CNR and LDI for each Likert score was calculated using the data for both systems as the mean extended CNR and LDI of all spheres with the corresponding Likert score across all SBRs and acquisition times. For each Likert score, the standard deviation was used as the error for each average extended CNR and LDI. This enabled the cross-validation of the extended CNR and LDI with the Likert scales, an effectiveness assessment of the three-point versus five-point Likert scale and comparison with the Rose criterion [34]. Spearman’s rank correlation coefficient ($\rho$) [35] was calculated for assessments of Likert scale against extended CNR and LDI.

**Table 2:** 3-point Lesion detectability Likert Scale with corresponding criteria

Likert score	Definition
0	A sphere indistinguishable from background noise
1	A detectable sphere, without a defined boundary
2	A clearly detectable sphere with a well-defined boundary

**Table 3:** 5-point Lesion detectability Likert Scale with corresponding criteria

Likert score	Definition
0	A sphere indistinguishable from background noise
1	A barely detectable sphere, potentially indistinguishable from background noise
2	A detectable sphere, without a defined boundary
3	A clearly detectable sphere with a defined boundary
4	A clearly detectable sphere with a well-defined boundary

The CNR can be calculated as

6 $$\begin{aligned} CNR = \frac{C_\text {on}}{N_m + N_a}, \end{aligned}$$

where C_on_ is the contrast and N_m_, N_a_ are the multiplicative and additive noise, respectively. Contrast is directly proportional to the mean number of detected photons within an image region, and therefore proportional to time, *t*. Since radiotracer decay and detection follow a Poisson distribution, multiplicative noise is proportional to the standard deviation of signal and therefore proportional to $\sqrt{t}$. Additive noise consists of two components, one that is proportional to *t* due to random counts and scattered photons, and another that remains constant over time, attributed to electronic noise. Therefore CNR can be expressed as

7 $$\begin{aligned} CNR = \frac{At^{\frac{1}{2}}}{1 + Bt^{\frac{1}{2}} + Ct^{-\frac{1}{2}}}, \end{aligned}$$

where A, B and C are constants. The CNR of the reconstructed images was investigated as a function of acquisition time and fitted to the model described by Eq. 7. This was used to estimate the acquisition time required to achieve a CNR value of 15, chosen based on the constraints imposed by the asymptotic nature of the curve, in both SiPM-based and PMT-based systems for all sphere sizes and low, medium and high radiotracer uptakes corresponding to SBRs of 4, 10 and 20. These uptakes were chosen based on tumour-to-background ratio ranges commonly seen in clinical practice [36]. The percentage reduction (PR) of acquisition time that can be achieved with the SiPM-based system versus the PMT-based system was calculated as

8 $$\begin{aligned} PR = 100 \times \Bigl ( 1-\frac{t_s}{t_{p}} \Bigl ), \end{aligned}$$

where $t_s$, $t_{p}$ are the estimated acquisition times to achieve the specified CNR of 15 for the SiPM-based and PMT-based systems, respectively. Uncertainty in PR was calculated by adding in quadrature the uncertainties for T_s_ and T_p_ that were calculated as the ratio between the standard deviation and the mean value of intensity of background voxels for the SiPM and PMT-based systems, respectively.

## Results

### Reconstructed images

**Fig. 2:**
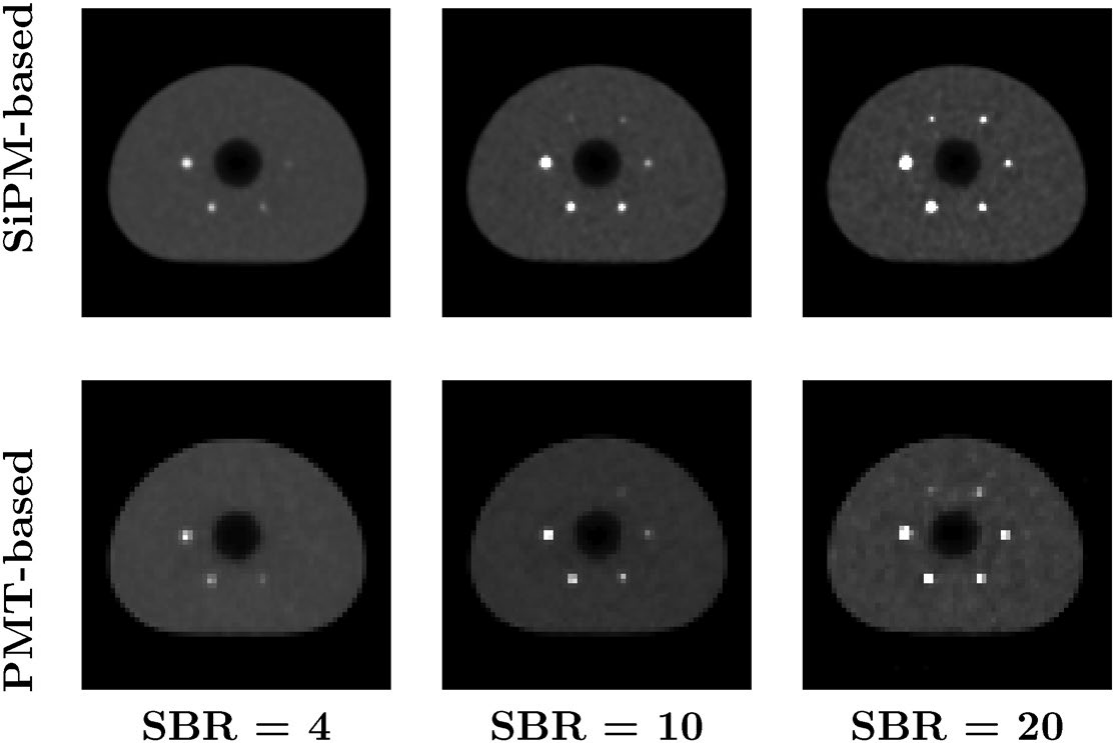
Reconstructed images for SBRs of 4 (left column), 10 (middle column), and 20 (right column) for the SiPM-based (top row) and PMT-based (bottom row) systems, for an acquisition time of 10 min

**Fig. 3:**
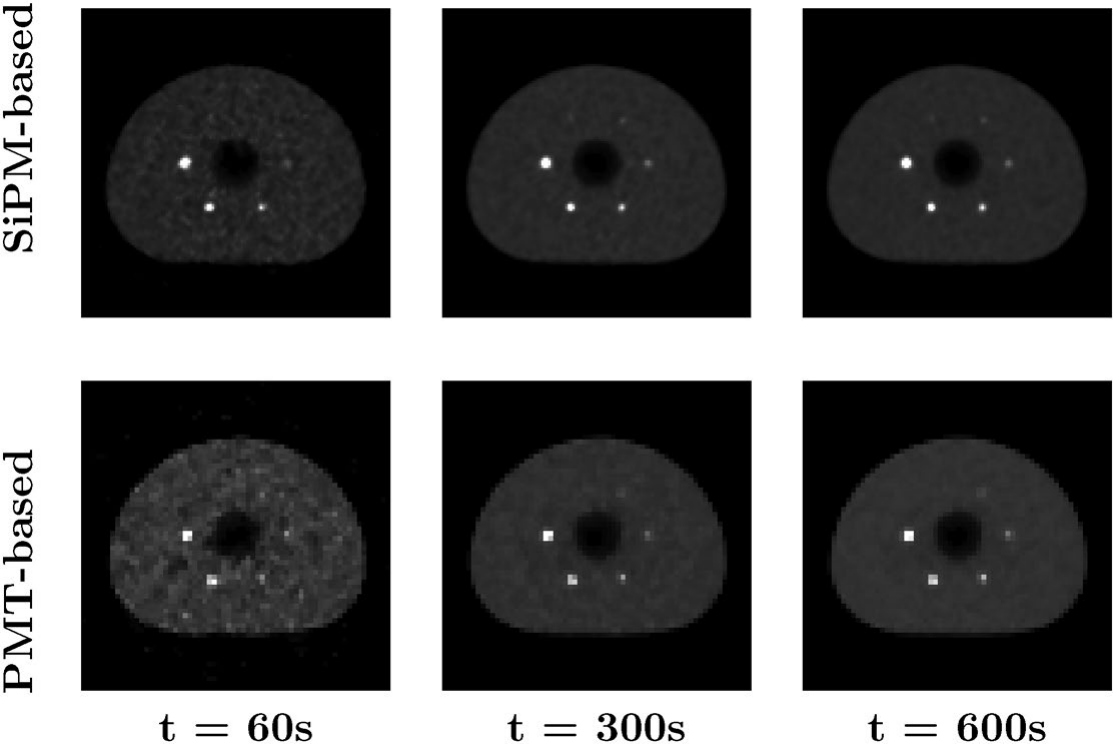
Reconstructed images for acquisition times of 60 s (left column), 300 s (middle column), and 600 s (right column) for the SiPM-based (top row) and PMT-based (bottom row) systems, for an SBR of 10

Reconstructed images for various SBRs and acquisition times are presented in Figs. 2 and 3, respectively, for both SiPM-based and PMT-based systems. We observe that the partial volume effect appears more pronounced in images acquired with the PMT-based system. The visibility of small lesions increases for both longer acquisition times and higher SBRs, but the latter factor has a more apparent impact. Additionally, a notable decrease in background noise can be observed with increasing acquisition time, as expected.

### Average contrast-to-noise ratio and lesion detectability index for each likert score

**Fig. 4:**
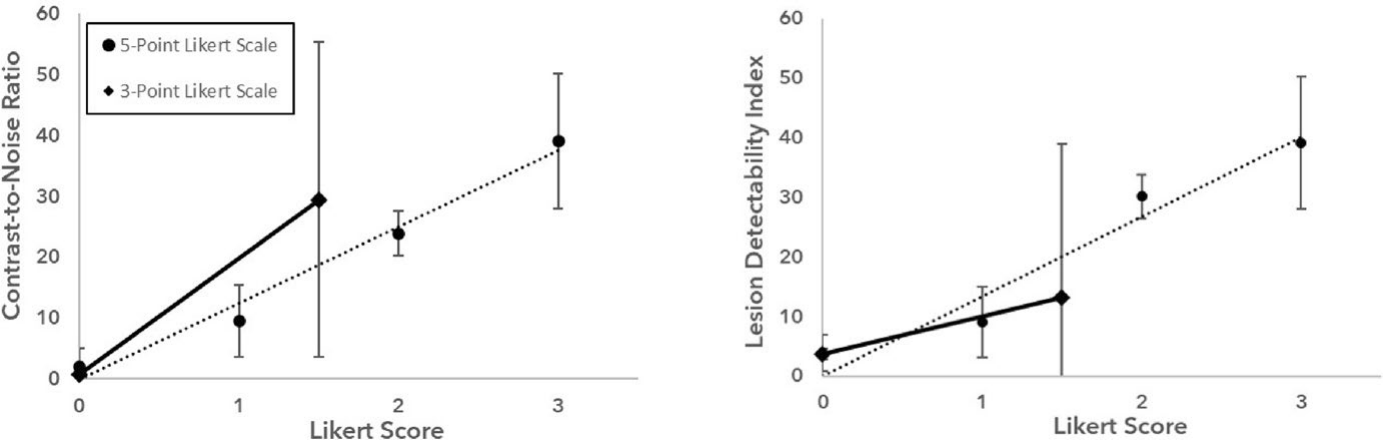
Average extended CNR and LDI for each Likert score of the three- and five-point Likert scales with the associated error bars representing the standard deviation of extended CNR and LDI for each Likert score. The three-point Likert scale scores were normalised to the five-point Likert scale scores for visualisation

Figure 4 presents the average extended CNR and LDI for each Likert scale score across both systems. For both the three- and five-point Likert scales, the last Likert values were omitted as these values have no upper bound on the CNR. A linear relationship between these quantities is seen, with R-values of above 0.98 between Likert values as a function of extended CNR and LDI across both Likert scales, suggesting high correlation between extended CNR and LDI against the human-observed Likert scale. Spearman’s rank correlations were 0.98 and 0.99 between the three-point Likert scale scores for extended CNR and LDI, respectively, and 0.97 and 0.98 between the five-point Likert scale scores and extended CNR and LDI, respectively. It is also observed that the minimum detectability level, defined as a Likert score of 0.5, was at a CNR of 10.4 and 6.3 for the three- and five-point Likert scales, respectively. This is consistent with the Rose criterion [34] which states that a lesion must have a CNR of above 5 to be detectable. It can be noted that the five-point Likert scale had a minimum CNR closer to the Rose criterion and had lower variance in average CNR and LDI values suggesting that the five-point scale provides more stable and consistent detectability assessments than the three-point scale.

### Contrast-to-noise ratio

**Fig. 5:**
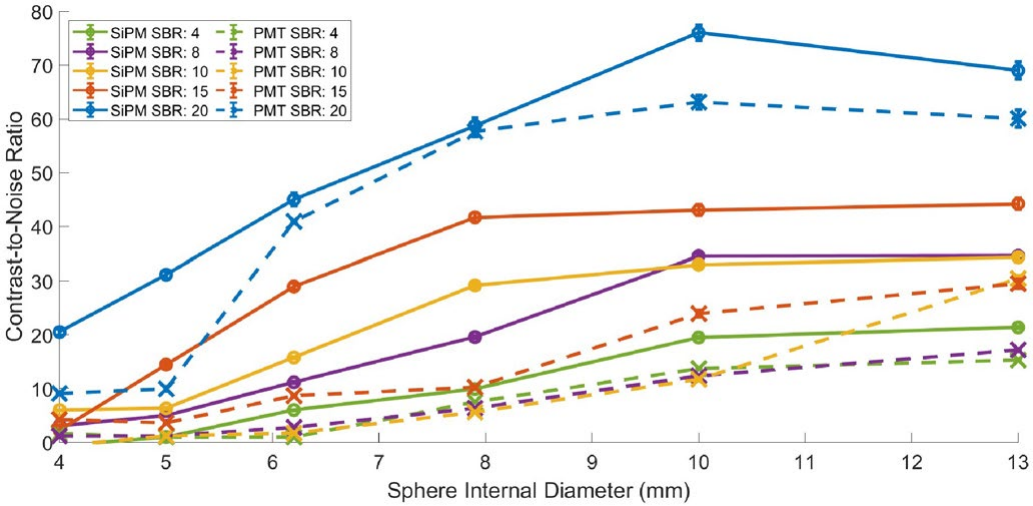
Contrast-to-noise ratio as a function of sphere internal diameter for various SBRs, for the SiPM-based and PMT-based systems, for an acquisition time of 10 min

**Fig. 6:**
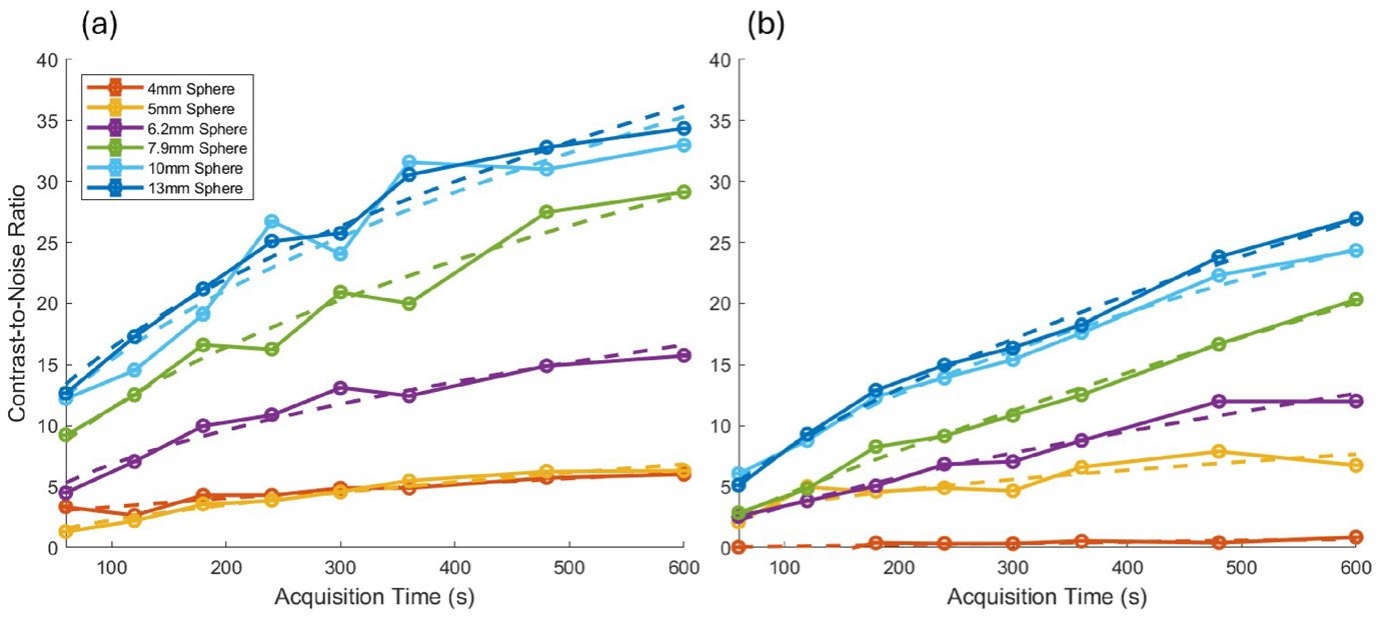
Contrast-to-noise ratio as a function of acquisition time for various sphere internal diameters, for an SBR of 10 for the SiPM-based (**a**) and PMT-based (**b**) systems. The dashed lines are fits using the model described by Eq. 7

The CNR as a function of sphere size is presented in Fig. 5, and the CNR as a function of acquisition time is presented in Fig. 6 for the SiPM-based and PMT-based systems. It is seen that the CNR decreases as the sphere size decreases for both systems, as expected. Both systems show comparable rates of CNR decline with sphere size, but the SiPM-based system maintains higher CNR levels across all sphere sizes and SBRs compared to the PMT-based system. Furthermore, the CNR for the PMT-based system approaches near-zero values at a much larger sphere size; for instance, at a sphere size of 6.2 mm and SBRs of 10, 8 and 4 the PMT-based system exhibits a CNR of around zero, whereas the SiPM-based system maintains a CNR above 5. Furthermore, we note that the difference between the curves for the SiPM-based and PMT-based systems varies with the SBR value and the range of values of the sphere size as these factors influence sphere detectability. A positive trend between acquisition time and CNR is also observed, as expected. Comparatively, the SiPM-based system exhibits consistently higher CNRs than the PMT-based system across the range of acquisition times used in this study.

**Fig. 7:**
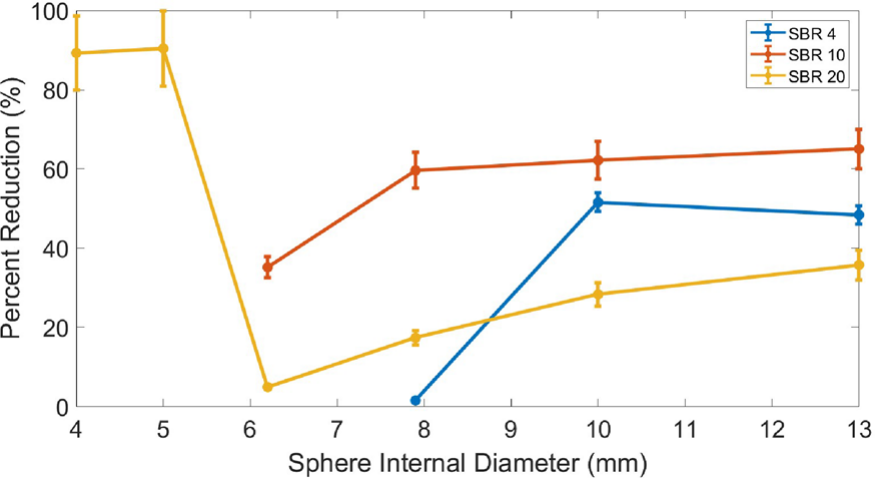
Percentage reduction in acquisition time for the SiPM-based system relative to the PMT-based system to achieve a CNR of 15 as a function of sphere internal diameter for SBRs of 4, 10 and 20

Table 4 presents the estimated acquisition times, calculated using the model described by Eq. 7, required to achieve a CNR of 15 for a specified uptake and sphere size for both PET systems, and the majority of coefficient of determination ($R^2$) for fitting the data to the model. Fitted values for coefficients A, B and C from the model presented in Eq. 7 are presented in Supplementary Table 1. The values of $R^2$ demonstrate that overall the model reliably predicts the acquisition time for sphere sizes larger than the pixel size of each system, and has better adherence to the data for smaller spheres for the SiPM-based system. The percentage reduction of acquisition time with the SiPM-based system relative to the PMT-based system (calculated using Eq. 8) is presented as a function of sphere size in Fig. 7. This shows that a reduction in acquisition time (or activity) can be achieved for the SiPM-based system whilst still maintaining a high level of detectability. Specifically, a reduction in acquisition time of between 1.6% and 89% can in principle be achieved depending on sphere size and activity uptake. The percentage reduction ultimately depends on system sensitivity and resolution. Across all SBRs, the SiPM-based system demonstrates superior performance due to its enhanced sensitivity, which increases the number of detected photons and subsequently reduces the impact of noise. Similarly, across all sphere sizes, the SiPM-based system is more performant due to its enhanced spatial resolution, which reduces the partial volume effect. Figure 7 indicates that the percentage reduction remains relatively stable for sphere sizes approximately between 10 and 13 mm across all three SBRs, and then decreases with the decrease of the sphere size, as the sphere internal diameter approaches the spatial resolution limit of both systems. With further decrease of the sphere size to below 6 mm, the percentage reduction exhibits an increase (for SBR 20). This increase occurs because the PMT-based system is unable to resolve objects of this size, while the SiPM-based system retains some degree of detectability, and is, thus, a reflection of the SiPM-based system’s ability to still detect objects that the PMT-based system cannot.

**Table 4:** Estimated acquisition time and $R^2$ values for various sphere internal diameters and SBRs. N/A indicates that a CNR above 15 is not achievable

SBR	Sphere size (mm)	PMT-based acquisition time (s) / $\mathbf {R^2}$ value	SiPM-based acquisition time (s) / $\mathbf {R^2}$ value
4	4.0	N/A	N/A
	5.0	N/A	N/A
	6.2	N/A	N/A
	7.9	1455/0.95	1432/0.94
	10.0	715/0.97	346/0.98
	13.0	576/0.99	297/0.99
10	4.0	N/A	N/A
	5.0	N/A	N/A
	6.2	781/0.99	506/0.98
	7.9	429/0.99	174/0.96
	10.0	270/0.99	102/0.92
	13.0	249/0.99	87/0.99
20	4.0	2256/0.56	241/0.96
	5.0	1750/0.88	167/0.98
	6.2	121/0.93	115/0.99
	7.9	92/0.96	76/0.99
	10.0	67/0.94	48/0.99
	13.0	81/0.98	52/0.99

### Lesion detectability

**Fig. 8:**
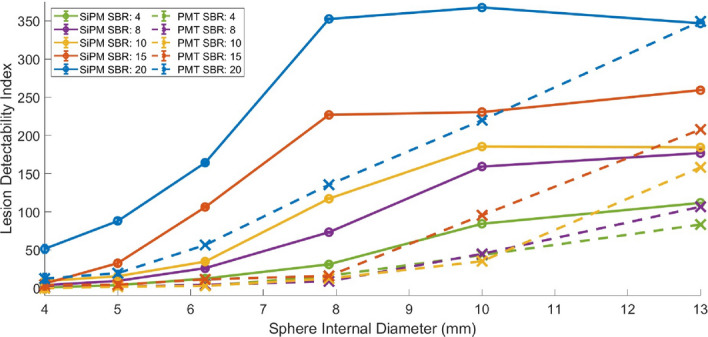
Lesion detectability index as a function of sphere internal diameter for various SBRs, for the SiPM-based and PMT-based systems, for an acquisition time of 10 min

**Fig. 9:**
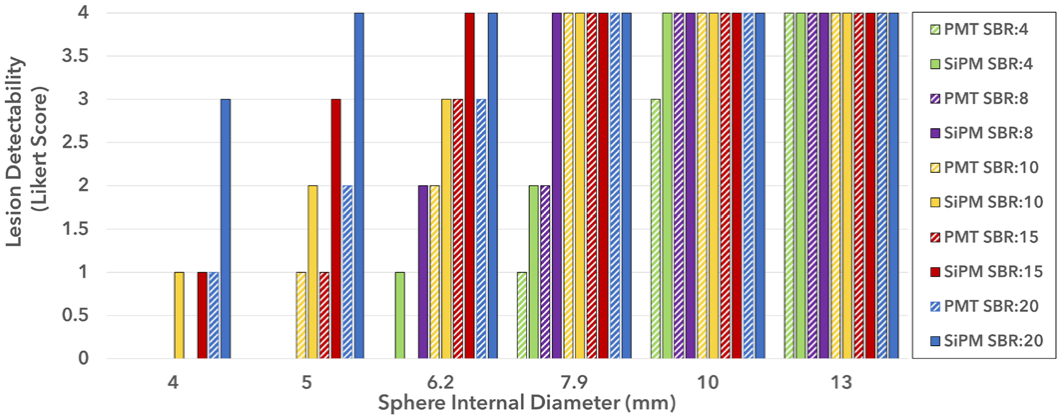
5-point lesion detectability Likert score as a function of sphere internal diameter for various SBRs for the SiPM-based and PMT-based systems, for an acquisition time of 10 min

Lesion detectability as a function of sphere size for the SiPM-based and PMT-based systems, using a lesion detectability index and 5-point Likert scale, is presented in Figs. 8 and 9, respectively. It can be seen that the SiPM-based system has superior lesion detectability across all sphere sizes and SBRs. Lesion detectability increases with SBR. The PMT-based system shows no detectability for SBRs below 20 for a sphere size of 4.0 mm, whereas the SiPM-based system maintains detectability for the 4.0 mm sphere for SBRs of 10 and above. It is also observed that the SiPM-based system has the same lesion detectability Likert score as the PMT-based system for some sphere sizes but for lower SBRs, indicating the ability of the SiPM-based PET to detect lesions with lower radiotracer uptake. For example, a sphere size of 7.9 mm has the same Likert score in both systems for an SBR of 8 versus 4 in the SiPM-based and PMT-based systems, respectively.

**Fig. 10:**
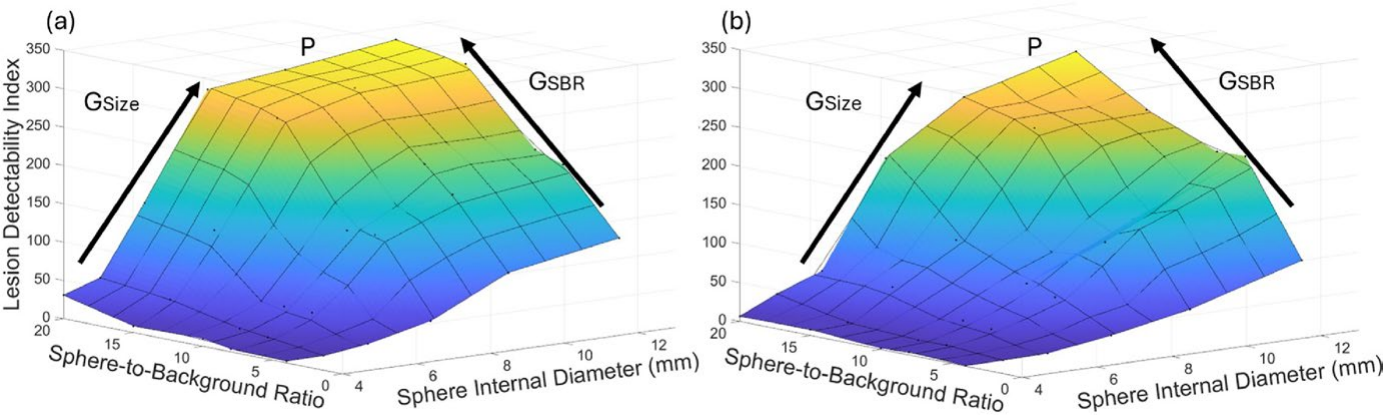
Lesion detectability index as a function of sphere internal diameter and SBR for an acquisition time of 10 min for the SiPM-based system (**a**), and PMT-based system (**b**). P, G_Size_ and G_SBR_ represent the apex, and the rate of increase in lesion detectability with sphere size and SBR, respectively

**Fig. 11:**
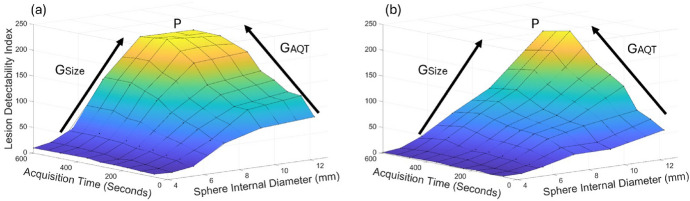
Lesion detectability index as a function of sphere size and acquisition time for an SBR of 10 for the SiPM-based system (**a**), and PMT-based system (**b**). P, G_Size_ and G_AQT_ represent the apex, and the rate of increase in lesion detectability with sphere size and acquisition time, respectively

Figures 10 and 11 present 3-dimensional plots of lesion detectability as a function of sphere size and SBRs or acquisition times, respectively. We note that the graphs for the SiPM-based system exhibit a more extensive flat region at the apex (P), indicating that maximum lesion detectability was attained over a broader range of SBR, sphere size, and acquisition time values, including lower values of these parameters. It can also be seen that lesion detectability increases at a greater rate with increasing SBR, sphere size, and acquisition time for the SiPM-based PET system.

### Contrast recovery

**Fig. 12:**
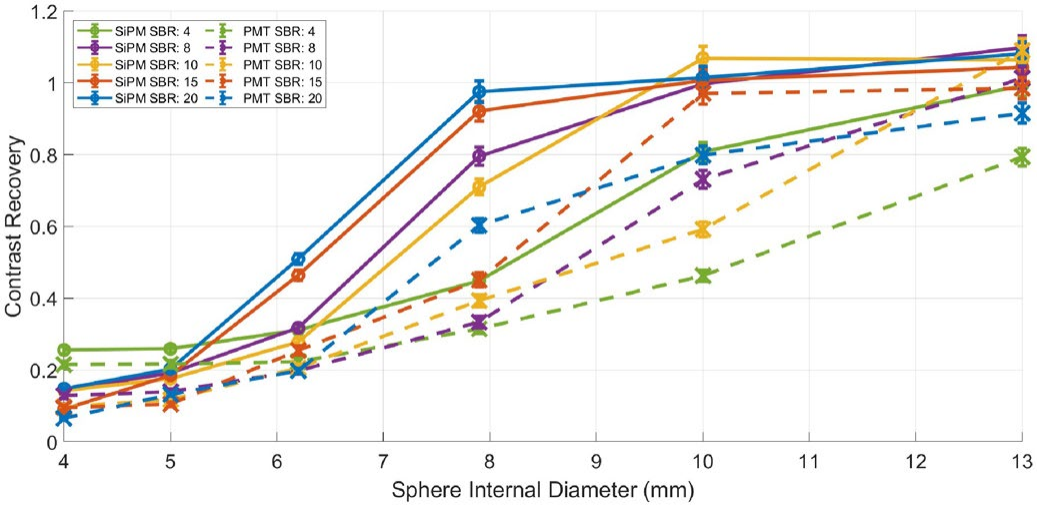
Contrast recovery as a function of sphere internal diameter for various SBRs, for the SiPM-based and PMT-based systems, for an acquisition time of 10 min

Contrast recovery as a function of sphere size for the SiPM-based and PMT-based systems and is presented in Fig. 12. As the sphere size decreases, contrast recovery decreases for both systems. However, the SiPM-based system consistently exhibits higher contrast recovery across all sphere sizes and SBRs. Both systems show a sharp decline in contrast recovery at a specific sphere size, but this decline occurs at smaller sphere sizes for the SiPM-based system. For instance, at an SBR of 10, a contrast recovery above one is maintained for sphere sizes above 10 mm for the SiPM-based system and only for sphere sizes above 13 mm for the PMT-based system. This is because the contrast recovery decreases as the size of an object approaches the spatial resolution of the system and the SiPM-based system has greater spatial resolution than the PMT-based system.

## Discussion

Notable differences in lesion detectability between the SiPM-based and PMT-based systems were observed, particularly for spheres with internal diameters smaller than 10.0 mm. These findings support the conclusion that SiPM-based PET systems offer improvements in detectability for smaller lesions. This is in contrast with the findings by Oddstig et al. that showed no significant difference in lesion detectability with even the PMT-based system exhibiting greater small-lesion detectability than the SiPM-based system in some cases. Similarly, Adler et al. reported no significant difference in lesion detectability for the SiPM-based system used in that work, although marginal improvements in lesion detectability were observed for shorter acquisition times and lower SBRs, which is consistent with the observations in our study. The increased small sphere detectability for the SiPM-based system across all acquisition times and SBRs seen in our study is likely due to its increased sensitivity of 20.8 cps/kBq versus 7.6 cps/kBq for the PMT-based system. This is also higher than the sensitivity of the SiPM-based systems used in Adler et al. and Oddstig et al. which were 5.6 cps/kBq and 13.8 cps/kBq, respectively [22]. To the author’s knowledge, no phantom or clinical study has investigated lesion detectability with a system of comparable sensitivity (of around 20 cps/kBq) to the SiPM-based system used in this work, against a lower-sensitivity system. System sensitivity has been cited as a significant factor in image quality and detectability with other studies [37]. Higher detector sensitivity increases the number of detected events, which allows for finer differentiation of structures and lesions, as the increased number of photons reduces the relative impact of background noise, thereby enhancing the overall CNR. Time of flight resolutions were different between the SiPM- and PMT-based systems used in this study (at 500 ps and 390 ps, respectively), which may have contributed to the increased lesion detectability found for the SiPM-based system. The SiPM-based system investigated in Oddstig et al., however, had an identical time of flight resolution to the SiPM-based system in our study, and still found no significant difference in lesion detectability for the SiPM-based system. This suggests that it is likely that the increased lesion detectability observed in our study, compared to previous studies [17, 18], can primarily be attributed to the superior sensitivity of the SiPM-based system used in our work; future work could investigate high-sensitivity PET systems to confirm this conclusion.

This study demonstrates a linear relationship between the CNR and Likert scale of lesion detectability. The positive correlation of Likert score with the CNR shows that the five-point Likert score used in this study accurately determines the detectability of lesions for phantom studies, but also incrementally shows changes in detectability more effectively than a three-point Likert scale. Additionally, the five-point Likert scale had less variance in average CNR values and was more consistent with the Rose Criterion.

This work shows that acquisition time can be significantly reduced with SiPM-based PET systems relative to the PMT-based system, with reductions of 48%, 65%, and 36% for SBRs of 4, 10 and 20, respectively, for a 13 mm sphere. However, acquisition time reduction is highly dependent on sphere size and activity uptake. Therefore, careful consideration of these factors is essential when planning a reduction in time or activity with SiPM-based systems. In comparison, previous studies using patient data and an acquisition time below 120 s showed a reduction in acquisition time of 75% [19], or suggested a reduction from 180-210 s/bed to 90 s/bed [20]. Although patient data is clinically relevant, it comes with limitations, such as a delay between FDG injection and scanning [20] or patient motion (impacting the attenuation correction) that may affect the results. These factors were not present in our study.

The results of our study are also consistent with that of previous studies [16] where a reduction in acquisition time by a factor of 0.52 was achieved for a one-to-one coupled SiPM system (as opposed to the many-to-one coupled system used in this study) and using Iodine-124 (as opposed to Fluorine-18 used in this study). In addition, our study considers the variability in acquisition time reduction with clinical factors such as lesion (sphere) size and SBR and therefore reports various reduction factors.

Our study demonstrates that the SiPM-based PET system maintains a contrast recovery value above one for smaller sphere sizes, indicating its ability to produce intensity distributions which more accurately correspond to the actual activity. This, combined with the results for CNR and lesion detectability, suggest that a review of image quality testing workflows and image quality phantoms may be required to assess the performance of modern PET systems, which has been indicated previously [31]. Based on the findings in this study, it is recommended to replace the current largest sphere of 37 mm in the NEMA/IEC PET Body Phantom with a sphere of around 8 mm internal diameter.

In this study, the performance of each PET scanner was investigated using a single scan across five different SBR values. A phantom study based on repeated scans have found a variation in the maximum SUV value of around 2.5% for an acquisition time of 10 min, and the variability in the mean SUV value of around 0% for all acquisition times investigated [38]. Given that our study was performed under controlled conditions with an identical phantom, employed an equivalent time-activity product , and the same reconstruction method was used for both acquisitions, the SUV variance between acquisitions is expected to be negligible and is not likely to influence the conclusions of this study.

We note that while this study used relatively high activity concentration and acquisition times, cited as factors to improve lesion detectability [31], these would improve lesion detectability for both systems. Also, the 16% difference in activity concentration in the spheres between the SiPM- and PMT-based systems was corrected by adjusting acquisition time to achieve an equivalent time-activity product. Previous studies [39, 40] have shown that equivalent image quality can be achieved even with far greater initial differences in activity concentration than this study when a time-activity product was used. For instance, a 1 min scan with a full dose is equivalent to a 2 min scan with a half dose for optimal clinical diagnosis [39], and FDG PET/CT images could maintain comparable image quality across full-dose, half-dose, and quarter-dose acquisitions when the lower administered activity is offset by longer scans [40]. The difference in activity concentration in our work falls within the range investigated by these studies and is therefore unlikely to have impacted our results.

The matrix and pixel sizes in this study differed between the two systems: the SiPM-based system used a 256 $\times$ 256 matrix with a pixel size of 2.7 mm, whereas the PMT-based system used a 128 $\times$ 128 matrix with a pixel size of 5.5 mm. Previous studies [41, 42] have shown that matrix and pixel size influences detectability, finding that smaller pixel sizes can significantly improve detection performance, and larger matrix sizes have been shown to increase lesion detectability and image quality. PMT-based PET systems have lower photon detection efficiency and poorer timing resolution [23], leading to increased noise and lower CNR when using larger matrix sizes. Therefore, smaller matrix sizes are required to maximise lesion detectability. Each system’s clinical protocol, which includes matrix and pixel sizing, was optimised to maximise lesion detectability. As this study aimed to investigate the benefits of SiPM-based PET - including lesion detectability - the matrix and pixel size was chosen based on the clinical protocol of each system to ensure that the maximum clinical lesion detectability of the SiPM-based system was assessed against the maximum clinical lesion detectability of the PMT-based system. Therefore, the difference in matrix and pixel size is not considered a limitation.

This study did not incorporate an additional source of activity outside the field of view (FOV), which can affect the count rates everywhere. However, in previous studies [43], activity outside the FOV was found to have relatively small effect (1.2%) on scatter fraction. While increasing out-of-FOV activity could result in a decrease of CNR [44], any potential decrease in CNR would affect both systems and would therefore be unlikely to affect the conclusions of our study.

## Conclusion

High sensitivity SIPM-based PET systems have superior lesion detectability compared to PMT-based PET systems, particularly for lesions smaller than 10 mm. Lesion detectability is enhanced further in SIPM-based PET systems for regions of lower activity concentrations. Scanning time can be reduced with new SIPM-based PET systems, but consideration must be taken for factors such as lesion size and radioisotope uptake. A five-point Likert scale is an effective measure for lesion detectability that also reflects incremental changes in detectability. A review of image quality testing workflows and image quality phantoms may be required to assess the performance of modern PET systems.

## Additional file

Supplementary file 1.

## Data Availability

The datasets used and analysed in this study are available from the correspondence author upon request.
