# Supplementary material for: Assessing small-lesion detectability and acquisition time optimisation in silicon-detector-Based PET: a phantom study
Source: EJNMMI Phys. 2025 Dec 28;13:13. doi: 10.1186/s40658-025-00821-9 (PMC12858686; doi:10.1186/s40658-025-00821-9)
Supplement: Supplementary file 1 [file 40658_2025_821_MOESM1_ESM.pdf]

**Target SBR**	**Filling Volume**	**SiPM AC (MBq/L)**	**PMT AC (MBq/L)**	**SiPM Actual SBR**	**PMT Actual SBR**
4	Sphere<br>BKG	16.00<br>3.93	17.51<br>4.30	4.07	4.07
8	Sphere<br>BKG	17.04<br>2.09	20.25<br>2.49	8.15	8.13
10	Sphere<br>BKG	19.64<br>1.81	21.29<br>2.09	10.85	10.19
15	Sphere<br>BKG	22.08<br>1.44	29.57<br>1.94	15.33	15.24
20	Sphere<br>BKG	29.20<br>1.44	34.63<br>1.66	20.28	20.86

Table 1: The activity concentrations (AC) within the sphere and background volumes for the SiPM-based and PMT-based systems, corresponding to each target sphere-to-background activity concentration ratio (SBR), along with the actual SBR that was achieved.

Sphere Size (mm)	Coef	SBR 4		SBR 10		SBR 20	
		PMT	SiPM	PMT	SiPM	PMT	SiPM
**4**	A	N/A	N/A	N/A	N/A	0.558	1.00×10^5
	B	N/A	N/A	N/A	N/A	0.0143	3.62×10^3
	C	N/A	N/A	N/A	N/A	4.12	7.59×10^5
**5**	A	N/A	N/A	N/A	N/A	1.14×10^4	9.54×10^3
	B	N/A	N/A	N/A	N/A	528	165
	C	N/A	N/A	N/A	N/A	4.10×10^5	7.86×10^4
**6.2**	A	N/A	N/A	0.936	2.30×10^4	3.31×10^6	28.3
	B	N/A	N/A	4.20×10^{-3}	1.05×10^3	4.54×10^4	0.288
	C	N/A	N/A	17.5	2.44×10^5	2.11×10^7	174
**7.9**	A	0.158	0.460	0.700	0.830	4.05×10^6	8.27
	B	-0.132	2.70×10^{-3}	-0.0183	-9.70×10^{-3}	3.34×10^4	0.0496
	C	-3.54	2.39	7.14	-1.88	2.19×10^7	29.6
**10**	A	1.37×10^4	2.56×10^4	0.896	10.1	8.99×10^6	6.50
	B	583	816	-7.40×10^{-3}	0.193	8.24×10^4	0.0232
	C	2.38×10^5	3.08×10^5	1.68	38.5	3.46×10^7	12.7
**13**	A	1.27	2.73	1.12	2.83	4.93×10^6	4.86
	B	0.0356	0.0546	-6.90×10^{-3}	0.0331	4.39×10^4	0.0144
	C	4.45	20.8	4.48	4.19	2.31×10^7	9.98

Table 2: Coefficient values A, B and C, fitted to the model presented in EQ.7 for sphere sizes and sphere-to-background activity concentration ratios (SBRs) for the PMT- and SiPM-based systems.
